# ST-Producing *E. coli* Oppose Carcinogen-Induced Colorectal Tumorigenesis in Mice

**DOI:** 10.3390/toxins9090279

**Published:** 2017-09-12

**Authors:** Peng Li, Jieru E. Lin, Adam E. Snook, Scott A. Waldman

**Affiliations:** 1 Department of Pharmacology and Experimental Therapeutics, Thomas Jefferson University, Philadelphia, PA 19107, USA; penglijennyli@gmail.com (P.L.); egerialin@gmail.com (J.E.L.); adam.snook@jefferson.edu (A.E.S.); 2 Department of Pathology, Immunology, and Laboratory Medicine, University of Florida, Gainesville, FL 32611, USA; 3 University of Illinois Chicago School of Medicine, Chicago, IL 60612, USA

**Keywords:** enterotoxigenic *E. coli*, heat-stable enterotoxins, azoxymethane, colorectal cancer, GUCY2C-cGMP axis, chemoprevention

## Abstract

There is a geographic inequality in the incidence of colorectal cancer, lowest in developing countries, and greatest in developed countries. This disparity suggests an environmental contribution to cancer resistance in endemic populations. Enterotoxigenic bacteria associated with diarrheal disease are prevalent in developing countries, including enterotoxigenic *E. coli* (*ETEC*) producing heat-stable enterotoxins (STs). STs are peptides that are structurally homologous to paracrine hormones that regulate the intestinal guanylyl cyclase C (GUCY2C) receptor. Beyond secretion, GUCY2C is a tumor suppressor universally silenced by loss of expression of its paracrine hormone during carcinogenesis. Thus, the geographic imbalance in colorectal cancer, in part, may reflect chronic exposure to ST-producing organisms that restore GUCY2C signaling silenced by hormone loss during transformation. Here, mice colonized for 18 weeks with control *E. coli* or those engineered *to* secrete ST exhibited normal growth, with comparable weight gain and normal stool water content, without evidence of secretory diarrhea. Enterotoxin-producing, but not control, *E. coli*, generated ST that activated colonic GUCY2C signaling, cyclic guanosine monophosphate (cGMP) production, and cGMP-dependent protein phosphorylation in colonized mice. Moreover, mice colonized with ST-producing *E. coli* exhibited a 50% reduction in carcinogen-induced colorectal tumor burden. Thus, chronic colonization with *ETEC* producing ST could contribute to endemic cancer resistance in developing countries, reinforcing a novel paradigm of colorectal cancer chemoprevention with oral GUCY2C-targeted agents.

## 1. Introduction

Enterotoxigenic *E. coli* (*ETEC*) remain a major public health issue, causing almost 1 billion illnesses and half a million deaths worldwide each year, with deaths mostly occurring in developing countries in children less than 5 years old [1,2,3]. *ETEC* is a heterogeneous classification of bacteria, comprising molecular subtypes of *E. coli* identified by their toxins that produce secretion. These include heat-labile enterotoxins (LT) which are structurally homologous to cholera toxin and induce cyclic adenosine monophosphate (cAMP) accumulation, and heat-stable enterotoxins (STa and STb) which induce cGMP accumulation [1]. Of these, STa (ST) is the predominant form associated with human disease, comprising 18 amino acids containing three intrachain disulfide bonds that provide the structural stability underlying its resistance to heat-induced denaturation [1].

ST is structurally homologous to the paracrine hormones guanylin (GUCA2A) and uroguanylin (GUCA2B) which activate the intestinal guanylyl cyclase C (GUCY2C) receptor [4]. However, compared to these endogenous hormones, which contain only two disulfide bonds, ST is resistant to proteolysis, isomerically stable, pH-insensitive, and has a higher receptor affinity resulting in excess GUCY2C activation in the small intestine leading to diarrhea. Binding of ST to the extracellular receptor domain of GUCY2C activates the cytoplasmic catalytic domain that converts GTP to cyclic GMP (cGMP) [4]. In turn, cyclic nucleotide accumulation activates cGMP-dependent protein kinase (PKG) which phosphorylates and opens the cystic fibrosis transmembrane conductance receptor (CFTR), a channel permeable to chloride and bicarbonate ions. Chloride ions flow down their electrochemical gradient into the intestinal lumen, leading to electrogenic sodium flux and water secretion resulting in secretory diarrhea. This pathophysiological mechanism is the basis for the development of the ST analog linaclotide (*Linzess*™) approved by the FDA to treat patients with chronic constipation and constipation-type irritable bowel syndrome [5].

Beyond intestinal secretion, the guanylin-GUCY2C paracrine axis comprises a tumor suppressing circuit whose dysregulation universally characterizes colorectal carcinogenesis across species [6,7]. Indeed, guanylin is one of the most commonly lost gene products in colorectal tumorigenesis and its loss is one of the earliest events in malignant transformation in mice and humans [6,8,9]. Loss of guanylin silenced GUCY2C producing intestinal epithelial dysfunction disrupting homeostatic mechanisms organizing the crypt-villus axis including proliferation, DNA damage sensing and repair, and metabolic programming, which contribute to tumorigenesis [10,11,12,13,14]. Further, silencing GUCY2C amplified intestinal tumorigenesis induced by carcinogens or genetic mutations in mice [10,11,12]. Moreover, GUCY2C ligand replacement opposed mutational or carcinogen-induced intestinal carcinogenesis in mice [9,12]. In that context, GUCY2C ligand replacement is emerging as a novel paradigm for colorectal cancer chemoprevention [15,16].

Colorectal cancer is the third leading cause of cancer and the fourth leading cause of cancer-related mortality in the world, with a geographic distribution primarily affecting patients in developed, compared to developing, countries [17,18,19,20,21]. The epidemiology of this disease remains incompletely understood, and its complexity reflects contributions of diet, lifestyle, comorbidities, environmental exposures, access to healthcare, and socioeconomic factors [17,18,19,20,21]. Interestingly, there is an unexplained inverse relationship between the incidence of colorectal cancer and *ETEC* infections [9,14]. Indeed, the age-adjusted incidence of colorectal cancer is lowest in under-developed countries where ETEC infections are highest [14,18,19,21]. The role of GUCY2C as a tumor suppressor and the loss of guanylin expression in tumor pathophysiology could represent one contributing factor to this inverse epidemiological association between colorectal cancer and *ETEC* infections, reflecting longitudinal exposure to ST-producing bacteria in developing countries. Here, we reveal for the first time that chronic colonization of mice with *E. coli* producing ST opposes the development of colorectal tumors induced by the carcinogen azoxymethane (AOM). These studies support the hypothesis of an environmental contribution to the disparity in colorectal cancer incidence in developed and developing countries, mediated by chronic exposure to ST-producing bacteria [14]. Moreover, it underscores the emerging paradigm of oral GUCY2C ligand replacement as a novel approach to the chemoprevention of colorectal cancer [9,11,15].

## 2. Methods and Materials

**Recombinant bacteria.** An ST-secreting construct containing the ST pro-peptide sequence [22] was generated using the pET101/D-TOPO ampicillin-resistant plasmid vector (cat# K10101; Thermo Fisher Scientific, Philadelphia, PA, USA). BL21, a chemically competent *E. coli* suitable for protein expression (New England Biolabs, Ipswich, MA, USA), was transformed using the recombinant ST-containing construct to generate ST-secreting *E. coli* [BL21(ST+)]. BL21 *E. coli* transformed with empty plasmid vector (no ST secretion) served as a negative control [BL21(ST−)].

**Mouse and bacterial colonization.** SWR/J mice (Jackson Laboratory, Bar Harbor, ME, USA), susceptible to AOM-induced colon carcinogenesis, received drinking water ad libitum containing ampicillin (1 g/L) for 1 week at 3 weeks of age, followed by oral gavage with bacteria in 200 μL PBS (OD = 1.0) at 4 weeks of age. Mice received water ad libitum containing ampicillin for the duration of the experiments to maintain intestinal bacterial colonization. Bacteria were isolated from mouse stool weekly starting at week 5 to week 18 [23] and stool weight was used to normalize the colony forming units for both BL21(ST−) and BL21(ST+). While each cohort initiated with 25 mice, 23 mice in the BL21(ST−), and 22 mice in the BL21(ST+), survived to the analytical endpoint (18 weeks of age).

**Enterotoxin quantification by EIA.** BL21(ST−) and BL21(ST+) isolated from mouse stool were maintained in Luria-Bertani (LB) broth and supernatants were collected for quantification of ST secretion. ST was quantified using the COLIST enzyme immunoassay (EIA) (Denka Seiken, San Jose, CA, USA).

**Azoxymethane (AOM) tumorigenesis.** Animal protocols were approved by the Thomas Jefferson University Institutional Animal Care and Use Committee (IACUC). Mice (6 weeks old) received intraperitoneal injections (12 mg/kg body weight) of AOM weekly for 6 weeks, and 12 weeks after the first injection were sacrificed and intestines examined for tumors.

**Immunoblot analysis.** Protein was extracted from tissues, lysed in Laemmli buffer supplemented with protease and phosphatase inhibitors (Roche; Sigma-Aldrich, Allentown, PA, USA). Lysates were analyzed by SDS-PAGE (NUPAGE 4–12% bis tris gel; Novex Life Technologies; Thermo Fischer Scientific, Philadelphia, PA, USA) and electrophoretically transferred to a nitrocellulose membrane (Novex Life Technologies). Membranes were blocked with 5% BSA in PBST (1X PBS and 1% Tween 20) and probed overnight with antibodies to p-ser157-VASP (#3111, 1:1000; Cell Signaling Technology, Danvers, MA, USA) and GAPDH (#2118, 1:5000; Cell Signaling Technology) followed by incubation with goat anti-mouse horseradish peroxidase (HRP)-conjugated and goat anti-rabbit HRP-conjugated secondary antibodies (1:50,000, Jackson ImmunoResearch, West Grove, PA, USA). Blots were developed in SuperSignal West Dura enhanced chemiluminescence substrate (Thermo Fischer Scientific). Relative intensity was quantified by densitometry using ImageJ and normalized to GAPDH. Results reflect the average relative intensity ± SD for ≥3 independent experiments.

**ST and control peptides.** ST1-18 was purchased from Bachem Co. (customer order; purity > 99.0%). ST was prepared by solid phase synthesis and purified by reverse phase HPLC, their structure confirmed by mass spectrometry by Bachem Co. (customer order; purity > 99.0%). ST activity was quantified by guanylate cyclase activation and secretion in the suckling mouse assay as described [24,25].

**Pathophysiological parameters.** Cyclic GMP was quantified by radioimmunoassay [10,12,13,14,26]. Stool water content was estimated by quantifying differences in stool weight before and after oven desiccation. Tumors were enumerated and their size quantified under a dissecting microscope in a blinded fashion. Tumor burden per animal was quantified by calculating the sum of the (diameter^2^) of individual tumors in each mouse [10,12]. All tumors from AOM-treated mice were histologically confirmed by a pathologist (PL) blinded to information for each case.

**Statistical analyses.** All data were analyzed using GraphPad Prism v6. Measurements were analyzed by ANOVA or Students t-test, unless otherwise indicated. Number of tumors per animal was analyzed by Poisson regression. Tumor burden and tumor size (mm^2^) in the continuous scale were analyzed by linear mixed models, with random effect of animal to control for multiple measures per animal. Tumor number, size and burden per animal were categorized and analyzed using Mantel-Hanzel (MH) exact chi-square tests for trend. *p* < 0.05 was considered statistically significant. Error bars depict 95% CI unless otherwise specified.

## 3. Results

### 3.1. Chronic BL21 Colonization and ST Production

**Figure 1:**
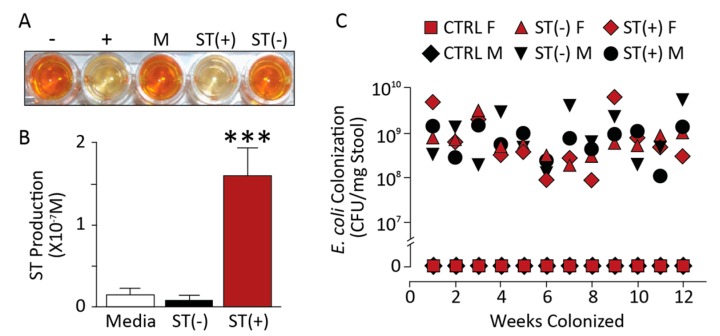
Colonization of mice with recombinant bacteria. (**A**,**B**) BL21(ST+) [ST(+)] produced heat-stable enterotoxin (ST) in vitro compared to BL21(ST−) [ST(−)] (*n* ≥ 3 cultures, each in triplicate). (**C**) BL21(ST+) [ST(+)] and BL21(ST−) [ST(−)] were recovered in stool from colonized mice for up to 18 weeks of age, compared to control mice [no recombinant bacteria (CTRL)]. CFU, colony forming units; F, female; M, male. *n* = 5 mice per group; error bars represent 95% CI; *** *p* < 0.001.

**Figure 2:**
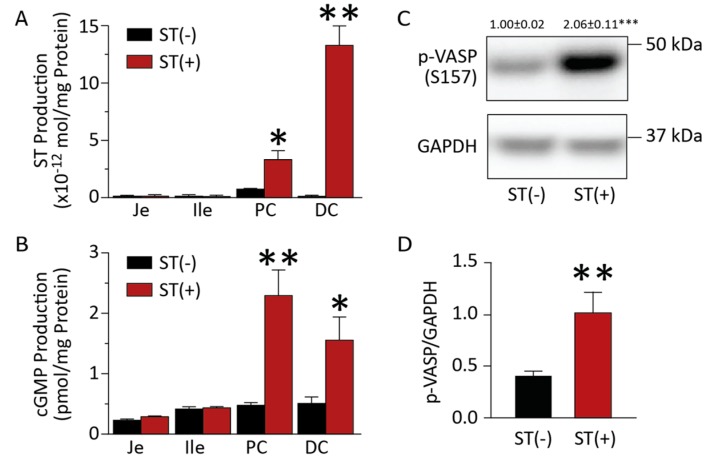
BL21(ST+) produce ST that activates GUCY2C downstream signaling in mouse intestine. (**A**) ST was quantified in intestinal content recovered from mice colonized for 3 weeks by BL21(ST+) [ST(+)], but not from BL21(ST−) [ST(−)]. (**B**) Cyclic GMP accumulated in epithelial cells recovered from intestinal segments of from mice colonized for 3 weeks by recombinant BL21(ST+) [ST(+)], but not from BL21(ST−) [ST(−)]. (**C**,**D**) VASP was phosphorylated in epithelial cells recovered from intestinal segments from mice colonized for 3 weeks by BL21(ST+) [ST(+)], but not by BL21(ST−) [ST(−)]. Je, jejunum, Ile, ileum, PC, proximal colon, DC, distal colon. *n* = 5 mice per group; assays were performed in triplicate; error bars represent 95% CI; * *p* < 0.05; ** *p* < 0.01; *** *p* < 0.001.

**Figure 3:**
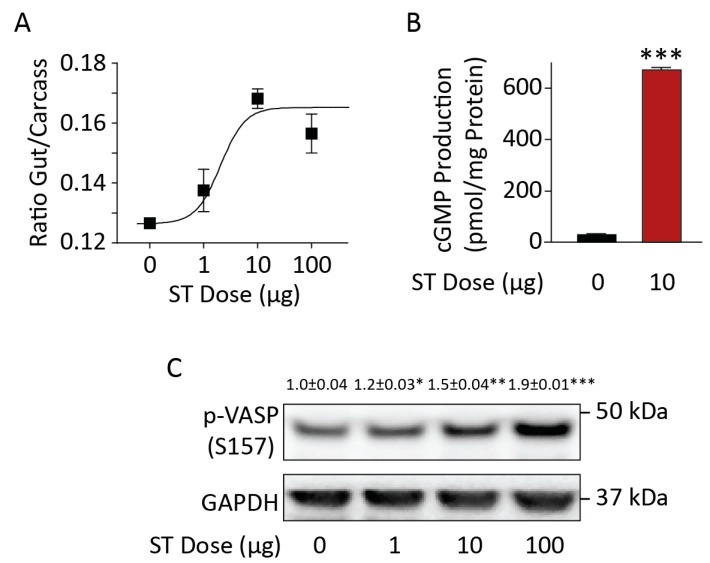
Synthetic ST mimicked the effects of ST-producing enterotoxigenic *E. coli* (*ETEC*) in mouse intestine. (**A**) Bioactivity of synthetic ST was verified using the suckling mouse assay. (**B**) Synthetic ST (10 μg) activated guanylyl cyclase C (GUCY2C) and cGMP production in epithelial cells recovered from the small intestine of adult mice. (**C**) Synthetic ST induced the phosphorylation of VASP in intestinal epithelial cells in a dose-dependent fashion. *n* = 5 adult mice per group; assays were performed in triplicate; error bars represent 95% CI; * *p* < 0.05; ** *p* < 0.01; *** *p* < 0.001.

**Figure 4:**
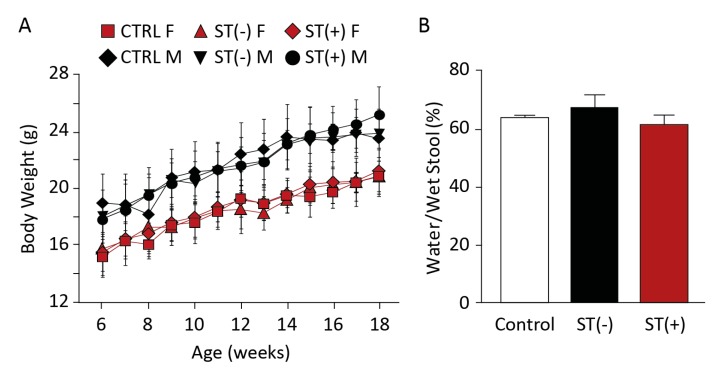
Mice chronically colonized by recombinant bacteria maintained normal growth. Mice chronically colonized with BL21(ST+) [ST(+)] or BL21(ST−) [ST(−)]. (**A**) experienced growth, quantified by weight gain, and (**B**) exhibited stool water content, that was comparable to control mice [no recombinant bacteria (CTRL, Control)]. *n* = 5 mice per group; assays were performed in triplicate; error bars represent 95% CI. F, female; M, male.

BL21(ST+) secreted immunoreactive heat-stable enterotoxin compared to BL21(ST−) (Figure 1A,B). These bacteria continuously colonized mice for 18 weeks, evidenced by their recovery in stool (Figure 1C). Colonization was limited to the colorectum, but not the small intestine (Figure 2A,B). In that context, BL21(ST+) secreted ST in colonized mice, activating GUCY2C and increasing cGMP accumulation (Figure 2B) and cGMP-dependent protein phosphorylation of VASP (Figure 2C), a canonical downstream target of cGMP signaling [27] (Figure 2C,D) in epithelial cells of the colon, but not the jejunum and ileum (data not shown). The effects of ST secreted by colonizing BL21(ST+) precisely mimicked those of synthetic biologically active ST (Figure 3A), which also activated GUCY2C and increased cGMP production (Figure 3B) and phosphorylation of VASP (Figure 3C) in intestinal epithelia. Mice colonized with BL21(ST+) and chronically exposed to ST exhibited normal growth characteristics, with weight gains that were comparable to mice colonized with BL21(ST−) (Figure 4A). Moreover, colorectal colonization with BL21(ST+) and chronic exposure to ST did not induce diarrhea, and water content of stool was comparable to that produced by mice chronically colonized with BL21(ST−) (Figure 4B).

### 3.2. BL21(ST+) Colonization Opposes Carcinogen-Induced Colorectal Tumorigenesis

**Figure 5:**
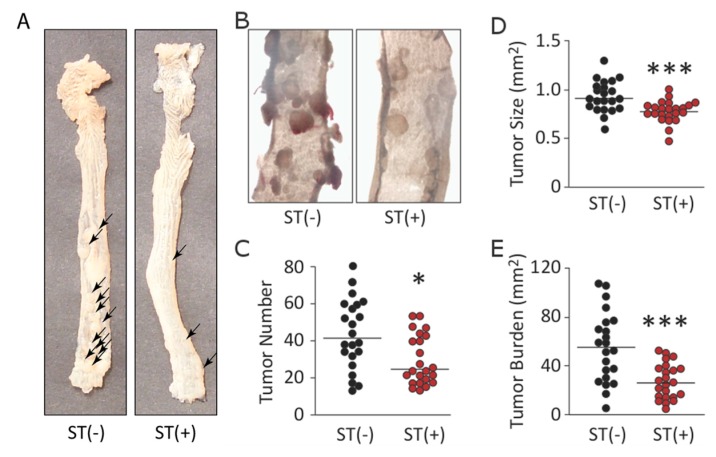
Chronic colonization with BL21(ST+), but not BL21(ST−), opposed azoxymethane (AOM)-induced tumorigenesis. (**A**,**B**) AOM specifically produced colorectal tumors (arrows) in mice. (**C**–**E**) Colonization of mice with BL21(ST+) [ST(+); *n* = 22] diminished the number (**C**) and size (**D**) of colorectal tumors, (**E**) reducing the tumor burden compared to mice colonized with BL21(ST−) [ST(−); *n* = 23]. Horizontal bars in (**C**–**E**) represent medians; * *p* < 0.05; *** *p* < 0.001.

AOM is a pro-carcinogen that is promoted in the liver to a DNA alkylating agent and mutagen, which induces intestinal tumors specifically in the colorectum [10,11]. AOM-induced tumors in mice chronically colonized with BL21(ST−) or BL21(ST+) (Figure 5A,B). However, colonization with BL21(ST+) inhibited tumor initiation, reflected by a lower number of tumors compared to mice colonized with BL21(ST−) (Figure 5C; *p* < 0.05). Also, colonization with BL21(ST+) inhibited tumor progression, reflected by significantly (*p* < 0.001) smaller tumors compared to mice colonized with BL21(ST−) (Figure 5D). Indeed, these effects on tumor initiation and progression resulted in a reduction in overall tumor burden of more than 50% in mice colonized with BL21(ST+) compared to mice colonized with BL21(ST−) (Figure 5E; *p* < 0.001).

## 4. Discussion

Diarrheal disease remains one of the leading causes of morbidity and mortality worldwide, responsible for 2.3 billion illnesses and 4% of all deaths annually [1,2,3]. ST−producing *ETEC* are a principal cause, with nearly a billion episodes, resulting in ~500,000 deaths, annually [28,29,30]. Importantly, the burden of diarrheal disease is concentrated in geographic regions that are under-developed, with negligible rates in industrialized countries [1,3,28,29,30]. While the clinical impact is greatest in particularly vulnerable populations, including children and the elderly, carriage rates of ST-producing *ETEC* in otherwise healthy individuals are substantial in endemic regions, reflecting chronic colonization in the absence of disease [1,3,28,29,30].

Colorectal cancer is the third most common cancer and the fourth most common cause of cancer death worldwide, accounting for >9% of cancer incidence and a million new cases annually [17,19,20]. Colorectal cancer is mainly a disease of industrialized countries [31] and developed countries account for >63% of all cases [20,32]. Indeed, incidence varies up to 10-fold between developed and under-developed countries, with >40 per 100,000 people in Westernized countries compared to <5 per 100,000 in under-developed geographic regions [17,20,33]. While there is a striking inverse epidemiological relationship between diarrheal diseases related to ST-producing *ETEC* and colorectal cancer, mechanisms contributing to this inverted linkage remain undefined [9,14].

ST-producing *ETEC* induce diarrhea by binding to GUCY2C, the intestinal isoform of membrane-bound guanylyl cyclase [4,24,34]. STs are an example of convergent evolution in which bacteria evolved a peptide that is a molecular mimic of the paracrine hormone guanylin, the endogenous ligand in the colorectum for GUCY2C [4]. Binding of endogenous (guanylin) or exogenous (STs) ligands to GUCY2C increases intracellular cyclic GMP (cGMP) [4,24,34,35,36,37]. In turn, GUCY2C-cGMP induces phosphorylation of the CFTR resulting in epithelial secretion, one mechanism by which bacteria induce diarrhea [4,38,39,40].

Beyond secretion, GUCY2C regulates intestinal epithelial homeostasis, including key component processes canonically disrupted in cancer [4,10,11,12,14,15]. Thus, guanylin loss expands the proliferating crypt compartment in colon, and silencing GUCY2C produces crypt hyperplasia by accelerating the enterocyte cell cycle [10,11,12,13,14,26,41]. Also, intestinal epithelia exhibit a metabolic gradient along the crypt-surface axis, where proliferating crypt cells generate ATP through glycolysis, while differentiated surface cells depend on oxidative phosphorylation [12]. Silencing GUCY2C imposes a glycolytic metabolic phenotype along the crypt-surface axis, with reduced mitochondrial content and function, increased glycolytic enzyme content, decreased oxygen consumption, and accumulation of lactate, recapitulating the Warburg metabolic phenotype in tumors [12]. Further, silencing GUCY2C increases DNA oxidation, double strand DNA breaks, mutations in tumor suppressor genes, and chromosomal instability [10,12].

In the context of this key role in epithelial homeostasis, guanylin is universally lost early in tumorigenesis, silencing GUCY2C, and this early hormone loss is a disease mechanism conserved in mice and humans [7,8,9,42,43]. Silencing GUCY2C signaling increases transcriptional programs driving tumorigenesis [10,11,12]. Indeed, eliminating GUCY2C expression amplified [10], while oral GUCY2C ligand reduced [9], tumors in mouse models mimicking human colorectal carcinogenesis. Further, transgenic guanylin expression that cannot be suppressed eliminated tumors in carcinogen-induced mouse models of colorectal cancer [11]. Taken together, these observations suggest a pathophysiological hypothesis that guanylin loss silencing GUCY2C signaling is an essential step in colorectal tumorigenesis [15]. Moreover, it suggests the correlative therapeutic hypothesis that oral GUCY2C ligand replacement may be a tractable approach to prevent colorectal cancer in patients [16].

This role for guanylin loss silencing GUCY2C in intestinal tumorigenesis suggests that the inverse epidemiological relationship between diarrheal disease and colorectal cancer could, in part, reflect chronic colonization in endemic areas with ST-producing *ETEC* [9,14]. In this model, chronic colonization with *ETEC* supplies exogenous ST that, in turn, reconstitutes GUCY2C signaling silenced by loss of the endogenous paracrine hormone guanylin, a universal step in tumorigenesis. Here, we demonstrate that mice continuously colonized with *E. coli* producing ST in the colorectum exhibit normal growth, without evidence of diarrheal disease, representing a model that recapitulates otherwise healthy patients in endemic areas chronically colonized with *ETEC*. Indeed, while these mice remain asymptomatic, ST is secreted into their intestinal lumens, binding to GUCY2C and stimulating cGMP production and signaling. Importantly, chronic colonization by ST-producing *E. coli* reduces the ability of the carcinogen AOM to initiate tumors (number), and drive their progression (size), diminishing tumor burden by >50%, compared to mice colonized by control *E. coli*.

Taken together, these observations demonstrate that chronic colonization with ST-producing *E. coli* reduces intestinal tumorigenesis induced by the carcinogen AOM. They support the hypothesis that the inverse epidemiological relationship between colorectal cancer and infectious diarrheal disease, at least in part, reflects a contribution of chronic colonization by *ETEC* that produce ST. In that context, our working hypothesis, yet to be directly tested, suggests that bacterially produced ST replaces guanylin lost during tumorigenesis, restoring GUYC2C signaling, which opposes tumor transformation [9,14,15]. Further, they support the pathophysiological hypothesis that colorectal cancer initiates as a disease of paracrine hormone insufficiency [15]. Moreover, they support the therapeutic hypothesis that colorectal cancer might be prevented by oral GUCY2C ligand replacement [9,11,15]. The immediate tractability of this therapeutic approach is underscored by the recent approval of linaclotide (*Linzess*™) and plecanatide (*Trulance*™), oral GUCY2C ligands approved for the treatment of chronic constipation syndromes [16,44].

## Abbreviations

AOM	azoxymethane
ETEC	enterotoxigenic *E. coli*
STs	heat-stable enterotoxins
LT	heat-liable enterotoxin
GUCY2C	guanylyl cyclase C
cGMP	cyclic GMP
PKG	cGMP-dependent protein kinase
CFTR	cystic fibrosis transmembrane conductance regulator
EIA	enzyme immunoassay
IACUC	institutional animal care and use committee
LB broth	Luria-Bertani broth
